# Annotations

**Published:** 1894-07-21

**Authors:** 


### ANNOTATIONS.

### Who Supervises the M.O.H.?

"Full many a flower is born to blush unseen, and waste its sweetness on the desert air," the poet Grey tells us. It has gradually dawned upon the mind of the present writer, as the result of the careful study of many annual reports, that the M.O.H. is sometimes a rare flower of this kind. Dr. J. C. McVail, Medical Officer of Health for the combined County Councils of Stirlingshire and Dumbartonshire, sends us his annual report for the past year, and we cannot help feeling that there is something radically wrong with our system of local government when the matter of which the report consists has no other publicity than that which is afforded by the cryptic pages of a blue book. If one first glances through the carefully-prepared table of contents, and then turns at random to any one of the subjects summarised, one feels that there is condensed in this report an amount of sanitary knowledge and broad common sense which ought to find an outlet in practical sanitary work on the broadest and most effective scale. In other words, one feels that there ought to be "bishops and overseers" for the supervision of medical officers of health, and that there are among existing officers of health not a few who would confer honour upon an episcopal office of this kind. It will be admitted by "those who know" that as things are at present the appointment and work of an M.O.H. is often a mere "playing at public sanitation." The sanitary schoolmaster has for some time been abroad; a little knowledge has been diffused, and a great deal of sentiment has been kindled; and this has had to be satisfied by the appointment of such a public official as the M.O.H. But should such "playing at sanitation" really satisfy a practical and intelligent people like ourselves? The time has come for leaving the ornamental stage behind and making our medical officers of health everywhere capable and everywhere actively efficient. This cannot be done except by supervision. There should be appointed supervising medical officers of health—"bishops," in short. It is for medical officers of health themselves to see the urgent practical necessities of their own service; and, seeing, to resolve that the country shall no longer be able to describe them and their offices as "salaried sinecurists and sinecures."

### Heat-stroke in England.

A good many cases of heat-stroke were noted in England during the recent hot weather, and some of them were unusually severe. It is too often taken for granted that since England ordinarily enjoys a temperate climate no special precautions need to be observed against the sun's heat. As a matter of fact, however, a few intensely hot days or weeks occurring in a climate which is ordinarily temperate are infinitely more dangerous to the "unseasoned" inhabitants than the months of hot weather to which the inhabitants of tropical and sub-tropical climates are accustomed. It is by no means generally known or suspected that an exposure of a very few minutes to a vertical midday sun may produce such a profound physiological shock as to kill the patient. Even in cases where death does not ensue there may be weeks of ill-health, and not seldom such a degree of permanent impairment of the brain power as to produce idiocy. Children are the persons most likely to suffer. Mothers and nurses need to be seriously warned. Too often mothers, merely because they are in the habit of sending children out at a certain hour of the day, send them out beneath a blazing sun to certain danger and frequent death. Children should not be sent out at all on very hot days between the hours of ten a.m. and four p.m. The average nurse is absolutely destitute of intelligence in matters of this kind, and very frequently the mother is little better. Routine is the bane of the ordinary woman's life. She will do what she is accustomed to do, and all the reasoning in the world will not prevent her. Then if her child is smitten to death, or almost to death by sunstroke, she wonders why Providence has sent her so terrible an affliction.

### What Epsom Does for Doctors.

Epsom College employs 220 district honorary secretaries to care for the interests of medical men in 220 principal towns of England. It provides 50 pensions for 50 decayed medical men or their wives. It receives 50 foundation scholars, the sons of deceased or poor medical men, and gives them the best education which England can furnish. It offers 9 perpetual presentations for female orphans of medical men. It gives annuities of £20 a year each to three Morgan annuitants. It furnishes the sons of ordinary medical men with a public school education of the highest class for £60 a year each. It secures for the profession a great public school, of which her Majesty the Queen is patron, the Duke of Abercorn president, the Bishop of Winchester visitor, and some of the most eminent persons in the ranks of the aristocracy and the learned professions are vice-presidents. It has Dr. Constantine Holman, J.P., for its indefatigable treasurer, and the Rev. T. N. Hart Smith, M.A., for its popular and successful head master. All this Epsom College is doing for doctors and their children. What are doctors and their children doing for Epsom College? This is a question which it behoves a good many medical men to consider, and give an answer to. A few medical men can answer the question with great satisfaction; they can say that, thanks to their energy and generosity, Epsom is what it is, and does what it does for the whole profession. The vast majority of doctors, however, would be obliged to confess that as yet they have done nothing at all for Epsom College. Then another question should be asked: Is Epsom College an institution of real and proved value to the medical profession? And if it is, ought not the profession, as a whole, to consider itself rather mean and "shabby" in leaving the support of the institution to the generosity of a few? The whole profession should support Epsom. It would give us the very sincerest satisfaction if we could hear that this little record of the work and value of Epsom College to the profession had induced a thousand doctors who had never helped the institution before to become annual subscribers forthwith.

